# Current concepts in the diagnosis and treatment of hypersensitivity pneumonitis: a narrative review

**DOI:** 10.36416/1806-3756/e20250394

**Published:** 2026-05-22

**Authors:** Guilherme das Posses Bridi, Isabela Maggioni Holz, Fabio Eiji Arimura, Ellen Caroline Toledo do Nascimento, Marcio Valente Yamada Sawamura, Ronaldo Adib Kairalla, Bruno Guedes Baldi

**Affiliations:** 1. Divisao de Pneumologia, Instituto do Coracao, Hospital das Clínicas, Faculdade de Medicina, Universidade de Sao Paulo - HCFMUSP - São Paulo (SP) Brasil.; 2. Núcleo de Pulmão, AC Camargo Cancer Center, São Paulo (SP) Brasil.; 3. Instituto D’Or de Pesquisa e Ensino, São Paulo (SP) Brasil.; 4. Departamento de Patologia, Faculdade de Medicina, Universidade de São Paulo, São Paulo (SP) Brasil.; 5. Instituto de Radiologia, Hospital das Clínicas, Faculdade de Medicina, Universidade de São Paulo - HCFMUSP - São Paulo (SP) Brasil.; 6. Núcleo de Tórax, Hospital Sírio-Libanês, São Paulo (SP) Brasil.; 7. Hospital do Coração, São Paulo (SP) Brasil.

**Keywords:** Alveolitis, extrinsic allergic/diagnosis, Alveolitis, extrinsic allergic/therapy, Lung diseases, interstitial

## Abstract

Hypersensitivity pneumonitis (HP) is an interstitial lung disease triggered by inhalation of a variety of environmental antigens. HP ranges from acute manifestations to progressive fibrosis and can be classified as nonfibrotic or fibrotic based on imaging and histopathological features. The pathophysiological mechanism of HP involves an exaggerated immune cell response and, in chronic stages, fibroblast activation leading to fibrosis. Diagnosis requires integrated assessment of exposure history, HRCT findings, BAL findings, and histopathological findings, as well as multidisciplinary discussion. In nonfibrotic HP patients, HRCT typically shows ground-glass opacities, centrilobular nodules, and mosaic attenuation, whereas, in fibrotic HP patients, HRCT often shows reticulation, traction bronchiectasis, and honeycombing. Typical histological features include cellular bronchiolitis, poorly formed granulomas, and interstitial fibrosis. Recent advances in imaging-including artificial intelligence-based pattern recognition-and investigation of biomarkers are improving precision and will likely contribute to the diagnostic investigation of HP. Management strategies depend on disease phenotype and severity, with antigen avoidance remaining the cornerstone of therapy. Immunomodulators such as corticosteroids, azathioprine, and mycophenolate may be used when there is evidence or suspicion of active inflammatory disease, whereas nintedanib is indicated for progressive fibrotic forms. Emerging phosphodiesterase 4B inhibitors have shown promise based on recent evidence. A greater number of comorbidities are observed in patients with fibrotic HP and are associated with worse survival, particularly pulmonary hypertension. Understanding genetic predisposition, comorbidities, and the transition from inflammation to fibrosis is essential to guide personalized treatment and improve outcomes. This review provides an update of pathophysiology, current diagnostic approaches, and therapeutic options, including emerging strategies.

## INTRODUCTION

Hypersensitivity pneumonitis (HP) is an immune-mediated interstitial lung disease (ILD) caused by repeated inhalational exposure to a wide variety of environmental antigens, including microbial agents, animal proteins, and chemical compounds. The disease results from a complex interaction between genetic susceptibility, environmental exposures, and immune response, leading to a broad clinical spectrum that ranges from reversible inflammation to progressive fibrosis. 

In recent years, significant advances have been made in the understanding of the pathophysiology and management of HP. The 2020 American Thoracic Society (ATS)/Japanese Respiratory Society (JRS)/*Asociación Latinoamericana de Tórax* (ALAT) Clinical Practice Guideline for the Diagnosis of Hypersensitivity Pneumonitis in Adults established a diagnostic approach based on clinical exposure history, HRCT patterns, BAL lymphocytosis, and histopathological features, emphasizing the role of multidisciplinary discussion to improve diagnostic confidence.^1^ A subsequent expert consensus statement published in 2021 by the American College of Chest Physicians (CHEST) provided additional insights.^2^

Despite these advances, the diagnosis of HP remains challenging because of the nonspecific nature of its clinical and radiological features; the overlap with other ILDs; and the difficulties in identifying potential causative antigens in many patients. Furthermore, treatment strategies are evolving in response to growing recognition of progressive fibrosing HP phenotypes, which may behave similarly to idiopathic pulmonary fibrosis (IPF). Recent trials have demonstrated the efficacy of antifibrotic agents such as nintedanib in patients with progressive fibrosing HP, leading to the inclusion of antifibrotics in treatment algorithms.^3^ ^,^ ^4^ This narrative review summarizes the current concepts in the pathophysiology, diagnosis, and management of HP, with emphasis on recent guidelines, evolving phenotypic classification, and therapeutic advances. 

## PATHOPHYSIOLOGY

The pathophysiology of HP is driven by a complex interaction between environmental exposures and host immune responses. In genetically susceptible individuals, repeated inhalation of organic or chemical antigens triggers both innate and adaptive immune pathways, leading to alveolitis, small airway inflammation, and, in chronic cases, progressive fibrosis. Early disease is dominated by a T cell-mediated response, whereas fibrotic HP involves a shift toward dysregulated repair mechanisms, epithelial injury, and fibroblast activation, resulting in excessive deposition of extracellular matrix and irreversible architectural distortion.^1^ ^,^ ^2^ ^,^ ^5^ ^-^ ^7^ In clinical practice, this evolution from an inflammatory to a fibrotic phenotype has relevant implications such as reduced responsiveness to immunosuppression and outcomes that resemble those seen in other progressive fibrosing ILDs. Highlighting these mechanistic shifts helps clinicians understand why early recognition and intervention are essential in modifying long-term prognosis. HP is triggered by the inhalation of a wide range of environmental antigens, referred to as “inducers.” These agents include microorganisms such as fungi, thermophilic actinobacteria, and atypical mycobacteria, as well as animal-derived proteins (e.g., feathers and bird droppings), plant particles, and low-molecular-weight chemical compounds capable of acting as haptens (Chart 1). Over 1,000 antigens have been reported as potential disease triggers.^1^ ^,^ ^2^ ^,^ ^6^ Accurate identification of the causative antigen is essential because antigen avoidance remains the cornerstone of both acute and chronic disease management.^5^ However, studies have shown that the antigen that is potentially responsible for HP is only identified in 30-50% of cases, which makes it difficult to control the disease.^8^ ^,^ ^9^ Repeated antigen exposure triggers dendritic cell-mediated activation of CD4^+^ T cells, promoting polarization toward Th1 and Th17 phenotypes and the subsequent release of IFN-γ, IL-17, and TNF-α. Additionally, immune complex-mediated injury and the presence of disease-associated autoantibodies in a subset of patients suggest that autoimmune mechanisms may perpetuate or aggravate inflammation even after antigen removal.^6^ ^,^ ^7^ The difficulty in identifying a causative antigen underscores the need for a comprehensive environmental and occupational history, sometimes requiring multiple interviews and information from family members. Moreover, because exposure often occurs at home rather than in the workplace, clinicians should maintain a high index of suspicion even in patients without an obvious occupational risk. When the antigen remains unknown, the disease tends to present more aggressively and with greater likelihood of chronicity, reinforcing the importance of environmental assessment as a central component of management. Several factors influence the risk of developing HP following antigen exposure. Environmental determinants include the type, intensity, frequency, and duration of exposure, with repeated or high-level inhalation of causative agents being strongly associated with disease onset. Host-related factors also play a crucial role; genetic susceptibility, including polymorphisms in genes related to immune regulation and telomere maintenance-including HLA alleles (HLA-DR and HLA-DQ), MUC5B promoter variants, TERT, TERC, PARN, and RTEL1-has been linked to increased risk and progression.^1^ ^,^ ^2^ ^,^ ^6^ Preexisting lung disease, age, and smoking status may further modulate both susceptibility and clinical phenotype, whereas immune system dysregulation, including aberrant T-cell responses and autoantibody production, may predispose certain individuals to persistent pulmonary inflammation and fibrotic remodeling.^5^ ^,^ ^7^ Recognition of these risk factors is essential not only for diagnosis but also for prognostic assessment. Patients with telomere-related mutations often exhibit more rapid progression and heightened vulnerability to treatment-related adverse effects, which may influence therapeutic choices. Similarly, identifying individuals with high-intensity or ongoing exposures allows targeted interventions-sometimes requiring multidisciplinary approaches. 

**Chart 1:** Environmental and occupational exposures, as well as possible sources, most commonly associated with hypersensitivity pneumonitis.*

Type of antigen	Specific antigens
Infectious	- Bacteria: *Acinetobacter* spp.; *Bacillus* spp.; *Klebsiella* spp.; *Pseudomonas* spp.; *Stenotrophomonas* spp.; *Streptomyces* spp.; endotoxin from pool water sprays and fountains; and nontuberculous mycobacterium (hospital water systems; humidifiers; air conditioning units; contaminated pool/spa water; agricultural and soil exposure; and wastewater environments) - Fungi/molds: *Aspergillus* spp.; *Cryptococcus* spp.; *Fusarium* spp.; and *Mucor* spp. (damp indoor environments; compost and soil; decaying vegetation; water-damaged buildings; and agricultural/greenhouse settings) - Yeasts: *Candida* spp. and *Geotrichum candidum* - Protozoa: Amoebae - Edible mushrooms: shiitake; *Pleurotus*; *Pholiota*; and *Agaricus* (mushroom cultivation farms; food-processing facilities; storage and packaging areas; and repeated culinary handling)
Organic	- Animal-derived proteins: animal fur dust; avian droppings and feathers; bats; cow’s milk; fish feed or meal; and shell proteins, such as oyster, sea snail, and mussels (bird breeding; poultry farms; feather bedding; barns and stables; dairy production; fish farming/processing; and seafood handling and processing) - Plant-derived proteins: grain flours (wheat; rye; oats; and maize); malt; legumes; paprika; alginate; argan cake; esparto dust; spinach; tiger nut; and wood (bakeries and grain mills; food-processing plants; spice handling; brewing and malting industries; woodworking; textile production with plant fibers; and agriculture involving grains, legumes, or nuts)
Inorganic	- Chemicals: acid anhydrides; acrylate compounds; copper sulfate; chloroethylene; isocyanates; tetrachlorophthalic acid; and sodium diazobenzene sulfate (manufacturing of plastics, resins, adhesives, or paints; chemical plants; spray-coating; pesticide handling; textile finishing; and metal-cleaning processes) - Pharmaceuticals: penicillins; cephalosporins; methotrexate; IFN-α; lenalidomide; pravastatin; venlafaxine; and temozolomide - Metals: cobalt; zinc; zirconium; and beryllium (metalworking and machining; welding; mining; battery production; and aerospace and ceramics industries)

*Adapted from Anwar et al.^9^

## EPIDEMIOLOGY

The prevalence and incidence of HP remain uncertain because of underdiagnosis and variability in diagnostic criteria across studies. Estimates suggest that HP accounts for approximately 2-15% of all ILDs, although this proportion varies widely depending on the geographic region, environmental exposures, and population studied.^10^ A large U.S. registry reported a prevalence of approximately 5% for HP among patients referred to specialized centers, with an annual incidence of 1.28 to 1.94 cases per 100,000 population and a prevalence ranging from 1.67 to 2.71 per 100,000 population. In the United Kingdom, the incidence of HP during the 1991-2003 period was reported to be 0.9 per 100,000 population,^11^ ^,^ ^12^ nearly twice that reported in a study conducted in Spain.^13^ However, in an ILD registry in India, HP was the most common cause of ILD, in 47.3%, followed by connective tissue disease-associated ILD (CTD-ILD), in 13.9%, and IPF, in 13.7%.^14^

In Latin America and other low- and middle-income regions, data remain limited but suggest that domestic exposures-particularly to birds and molds-are major contributors to the disease burden.^6^ ^,^ ^15^ In a recent meta-analysis involving patients with occupational HP, a prevalence of 16.71% was reported for South America in comparison with a prevalence of 15.19% for Asia and of 8.52% for North America.^16^ In Brazil, only one study evaluated the incidence of HP; of a total of 1,406 patients assessed for ILDs, 23% were diagnosed with HP, making it the second most common diagnosis after CTD-ILD (in 27%).^17^

## CLINICAL PRESENTATION

Symptoms of HP are often nonspecific and overlap with other ILDs, contributing to diagnostic delay.^7^ Common manifestations include progressive exertional dyspnea, nonproductive cough, and fatigue, often associated with unintentional weight loss in chronic cases. Fever, chills, malaise, and chest tightness are more frequently observed in patients with ongoing high-level exposures, particularly in those with nonfibrotic HP. Physical examination findings are usually limited to inspiratory crackles; digital clubbing is rare but may appear in patients with advanced fibrotic disease.^1^ ^,^ ^2^ ^,^ ^18^ Squawks may be present in patients with airway involvement (bronchiolitis).^19^ Patients with advanced disease, mainly those with hypoxemia and group 3 pulmonary hypertension (PH), may present with signs of right-sided heart failure.^20^

The current classification of HP focuses on nonfibrotic and fibrotic phenotypes,^21^ as shown in Chart 2. The nonfibrotic phenotype reflects an early, potentially reversible stage linked to recent antigen exposure, typically showing BAL lymphocytosis and a favorable prognosis.^22^ ^,^ ^23^ In contrast, fibrotic HP represents an advanced, irreversible stage with persistent inflammation, epithelial injury, and fibrotic remodeling, which are often due to delayed diagnosis or unrecognized exposure and poor response to antigen avoidance, with survival similar to IPF.^24^ HRCT commonly reveals a “three-density pattern,” centrilobular nodules, and air trapping, whereas BAL lymphocytosis is less common and shows only a weak negative correlation with the extent of fibrosis.^25^

**Chart 2:** Characteristics of and major differences between fibrotic and nonfibrotic hypersensitivity pneumonitis.

Feature	Nonfibrotic HP	Fibrotic HP
Clinical presentation	- Subacute or acute onset of cough, dyspnea, fever, and flu-like symptoms - Often reversible with exposure removal and/or corticosteroids - Favorable prognosis^1^ ^,^ ^2^	- Insidious onset, chronic dyspnea, and cough - Less clear temporal relation with antigen exposure - Potentially progressive and irreversible course - Poorer prognosis^1^ ^,^ ^2^
Pulmonary function	- Normal or mild restriction; DL_CO_ may be mildly reduced but improves with treatment - Signs of small airway involvement may be present^5^ ^,^ ^18^ ^,^ ^30^	- Restrictive pattern with reduced FVC and DL_CO_; progressive decline over time^5^ ^,^ ^18^ ^,^ ^30^
HRCT findings	- Typical: Diffuse GGOs with profuse centrilobular nodules and marked air trapping; diffuse distribution - Compatible: GGOs and mosaic attenuation without clear nodules; lung cysts; airspace consolidation; and diffuse distribution - Indeterminate: Nonspecific or limited findings that do not meet criteria for typical or compatible^1^ ^,^ ^2^	- Typical: Fibrosis with small airway disease (mosaic attenuation and air trapping) and centrilobular nodules, three-density pattern, and random distribution both axially and craniocaudally - Compatible: Peribronchovascular fibrosis indicative of small airway disease; and atypical distribution. - Indeterminate: Fibrotic changes lacking key HP features or overlapping with other ILDs (e.g., UIP, organizing pneumonia, and fibrotic NSIP pattern)^1^ ^,^ ^2^
Bronchoalveolar lavage	- Marked lymphocytosis (> 30-40%)^22^	- Lymphocytosis may be present but often less pronounced (< 20-30%)^22^
Histopathology	- HP: Cellular bronchiolitis, poorly formed granulomas, or interstitial lymphocytic infiltrates without established fibrosis - Probable HP: Bronchiolocentric interstitial pneumonia, cellular NSIP-like pattern, or lymphocyte-predominant infiltrates - Indeterminate for HP: Peribronchiolar metaplasia without other features to suggest fibrotic HP; organizing pneumonia pattern; or cellular NSIP pattern^1^ ^,^ ^2^ ^,^ ^5^	- HP: Chronic fibrosing interstitial pneumonia, airway-centered fibrosis, poorly formed nonnecrotizing granulomas, or fibrotic NSIP-like pattern - Probable HP: Chronic fibrosing interstitial pneumonia, airway-centered fibrosis, or organizing pneumonia pattern - Indeterminate for HP: Chronic fibrosing interstitial pneumonia, organizing pneumonia pattern, or fibrotic NSIP-like pattern^1^ ^,^ ^2^ ^,^ ^5^

GGOs: ground-glass opacities; HP: hypersensitivity pneumonitis; ILD: interstitial lung disease; NSIP: nonspecific interstitial pneumonia; and UIP: usual interstitial pneumonia.

It is clinically essential to distinguish between fibrotic and nonfibrotic phenotypes because they directly influence management decisions. Nonfibrotic HP tends to have greater potential for recovery when exposure is eliminated promptly, whereas fibrotic HP behaves as a chronic ILD with limited reversibility. This paradigm shift toward phenotype-driven assessment reflects the broader trend in ILD management, in which radiological patterns, functional decline, and disease trajectory now guide therapy more strongly than do single test results. Moreover, recognizing fibrotic HP at an early stage may prompt timely referral to ILD experts; consideration of antifibrotic therapy; and closer surveillance for complications such as PH. 

Patients with fibrotic HP frequently experience a progressive course similar to IPF in a context consistent with progressive pulmonary fibrosis (PPF), with higher rates of hospitalization, oxygen therapy dependence, and mortality in comparison with those seen in patients with nonfibrotic HP.^24^ ^,^ ^26^ Moreover, exposure cessation, which may lead to symptom improvement in patients with nonfibrotic HP, often has limited effect once extensive fibrosis is established.^27^ Chart 2 summarizes the main findings and differences between fibrotic and nonfibrotic HP. 

## DIAGNOSTIC APPROACH

The diagnosis of HP remains a clinical challenge because of the heterogeneous presentation of the disease and the overlapping features with other ILDs. A confident diagnosis typically requires integration of multiple domains, including a detailed clinical history with exposure assessment, radiological evaluation, BAL findings, histopathology, and, if available, multidisciplinary discussion. 

### Antigen exposure

A thorough evaluation of environmental and occupational exposures is a cornerstone in the diagnostic approach to HP (Chart 1). Identifying the inciting antigen is essential not only for confirming the diagnosis but also for guiding management, given that antigen avoidance is the most effective intervention to prevent disease progression.^24^ ^,^ ^26^ ^,^ ^27^ Because exposures may change over time or new sensitizing agents may be introduced, a structured exposure questionnaire should be applied at every clinical visit.^1^ ^,^ ^2^ ^,^ ^8^ ^,^ ^24^ This systematic reassessment increases the likelihood of identifying ongoing or previously unrecognized exposures, thus ensuring a more accurate diagnosis and better long-term outcomes. 

### Radiological patterns

**Figure 1:**
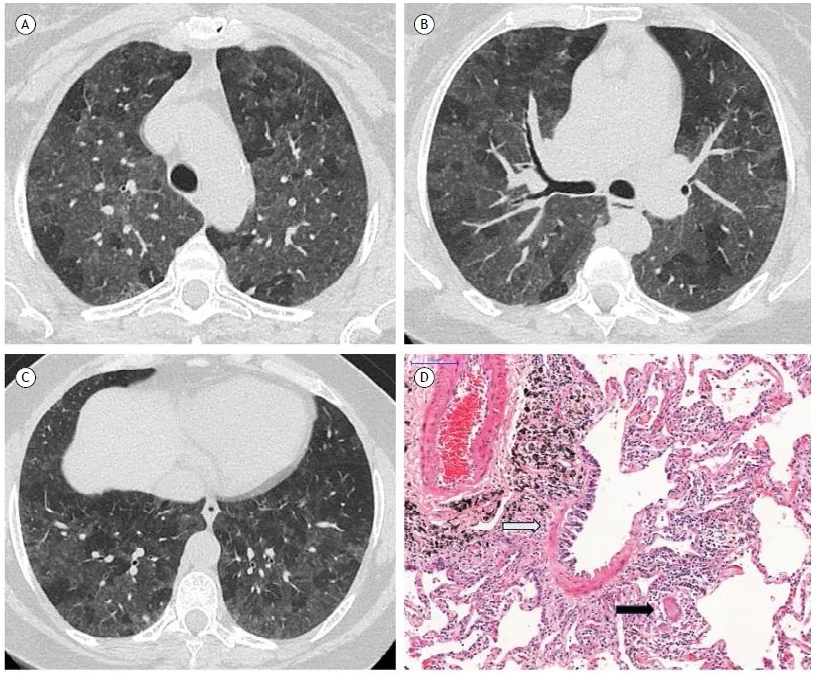
In A, B, and C, axial CT scan showing a typical pattern of nonfibrotic hypersensitivity pneumonitis, with centrilobular micronodules and areas of air trapping. In D, histopathology of nonfibrotic hypersensitivity pneumonitis. Mild lymphocytic inflammation of the bronchiolar wall (white arrow) with a poorly formed granuloma (black arrow); the inflammatory process extends into the interstitium (H&E; magnification, ×100).

In patients with nonfibrotic HP, HRCT typically reveals a constellation of findings reflecting airway-centered inflammation and parenchymal heterogeneity. The most characteristic features include ground-glass opacities, mosaic attenuation with air trapping, and centrilobular nodules, often with a diffuse distribution (Figure 1). The three-density pattern, previously referred to as the headcheese sign, is a characteristic HRCT finding in patients with HP and reflects the coexistence of distinct pathological processes within the same lung. It is best appreciated on inspiratory HRCT scans, on which three different attenuation areas appear sharply demarcated: normal appearing lung; ground-glass opacities representing active inflammation or fibrotic infiltration; and hypoattenuated regions related to small airway disease and air trapping.^1^ ^,^ ^2^ ^,^ ^5^ ^,^ ^7^ ^,^ ^18^ These radiological patterns, especially in a compatible clinical context, are critical for differentiating nonfibrotic HP from other inflammatory ILDs. 

**Figure 2:**
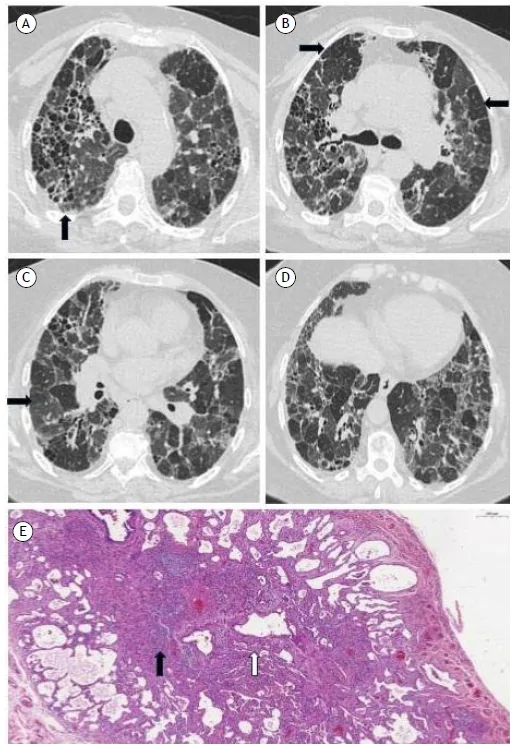
In A, CT scan showing areas of bronchiectasis and architectural distortion with a bronchiolocentric predominance (arrow). In B, areas of air trapping (arrows). In C, axial CT scan showing areas of normal lung (arrow) interspersed between affected lung and regions of air trapping. In D, the typical “three-density pattern.” In E, histopathology of fibrotic hypersensitivity pneumonitis. Septal and peribronchiolar fibrosis are present (white arrow), accompanied by mild lymphocytic inflammation and areas of peribronchiolar metaplasia (black arrow; H&E; magnification, ×100).

In patients with fibrotic HP, the most common radiological features include reticular opacities, traction bronchiolectasis, and varying degrees of honeycombing, often with a diffuse distribution (Figure 2). According to the 2020 ATS/JRS/ALAT guideline,^1^ HRCT findings in patients with fibrotic HP can be further categorized into typical, compatible, or indeterminate patterns (Chart 2). 

In addition to their diagnostic value, the extent and distribution of fibrosis on HRCT have important prognostic implications.^28^ ^,^ ^29^ In a study involving patients with HP and IPF, those with non-honeycomb fibrotic HP had better survival than did those with IPF (median, > 7.95 years vs. 5.20 years), although both groups experienced a decline in FVC. In contrast, the presence of honeycombing-whether in fibrotic HP or IPF-is associated with similar poor outcomes, with a median survival of approximately 2.8 years and marked FVC deterioration, underscoring the prognostic significance of radiology-driven subgroups.^30^

### Histopathological features

Lung biopsy plays an important role in the diagnostic evaluation of HP, particularly when clinical history, antigen exposure, and radiological features remain inconclusive after multidisciplinary discussion.^1^ ^,^ ^2^ Although surgical lung biopsy has traditionally been considered the gold standard, transbronchial cryobiopsy has emerged as a less invasive alternative, showing promising diagnostic yield with lower morbidity in specialized centers. It should be noted that cryobiopsy is currently not widely available.^31^ The decision to perform a lung biopsy should be individualized. 

Histopathologically, HP is characterized by a triad of airway-centered inflammation, poorly formed nonnecrotizing granulomas, and chronic interstitial infiltrates. Nonfibrotic HP typically shows bronchiolocentric lymphocytic inflammation, organizing pneumonia, and multinucleated giant cells (Figure 1).^1^ ^,^ ^2^ ^,^ ^7^ ^,^ ^32^ In fibrotic HP, overlapping features with other fibrosing ILDs, particularly usual interstitial pneumonia, often complicate diagnosis. Distinguishing findings include airway-centered fibrosis, bridging fibrosis, and a bronchiolocentric distribution of lesions (Figure 2). Typical fibrotic HP requires the presence of chronic interstitial fibrosis with airway-centered fibrosis and poorly formed granulomas. In contrast, the absence of granulomas in the presence of airway-centered fibrosis and distribution that is consistent with fibrotic HP are findings that support a diagnosis of probable fibrotic HP.^1^ ^,^ ^2^ According to international guidelines, lung biopsy should not be performed routinely, given its invasive nature and the increasing diagnostic accuracy of HRCT and antigen identification; lung biopsy should be reserved for cases in which diagnostic uncertainty persists and histopathology is expected to have a significant impact on treatment decisions.^1^ ^,^ ^2^ ^,^ ^5^ ^,^ ^33^ ^,^ ^34^

**Figure 3:**
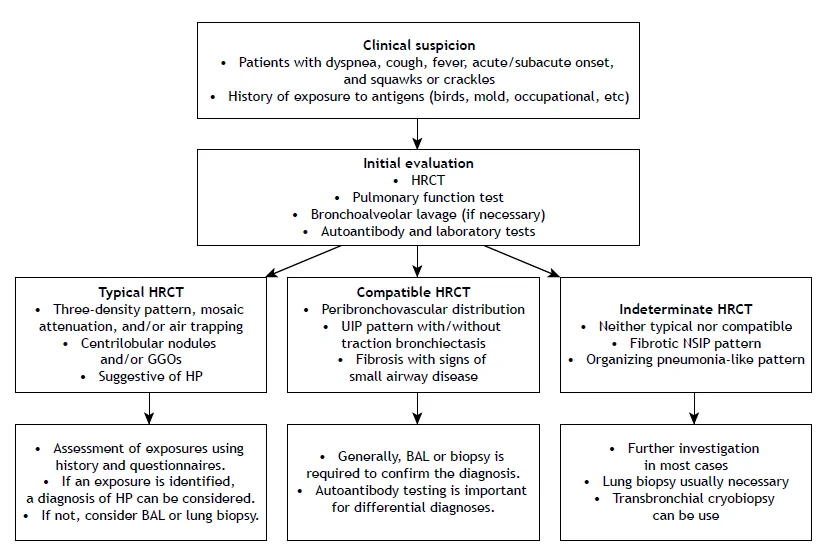
Flow chart for the diagnosis of hypersensitivity pneumonitis.* GGOs: ground-glass opacities; HP: hypersensitivity pneumonitis; UIP: usual interstitial pneumonia; and NSIP: nonspecific interstitial pneumonia. *Adapted from Raghu et al.^1^

In a recent prospective study including patients with a typical fibrotic HP pattern on HRCT, nearly one third had a probable or possible exposure; another third had no identifiable exposure; and 22% were reclassified as having CTD-ILD after multidisciplinary discussion. Patients without an identifiable exposure exhibited a greater decline in pulmonary function and shorter transplant-free survival; notably, 14% of those patients developed positive autoimmune serologies or clinical features of CTD during follow-up.^35^ In parallel, a recent viewpoint proposed revisiting the nomenclature of HP (Figure 3), emphasizing the recognition of bronchiolocentric interstitial pneumonia patterns on imaging and histopathology, and underscoring the need to refine HRCT and histopathological criteria to better reflect the central airway-based pathophysiology that distinguishes HP from other ILDs.^36^ ).

### International guidelines

Recent guidelines emphasize the importance of multidisciplinary integration, combining clinical, radiological, and histopathological data to establish a diagnosis. Comparative studies and critical reviews have shown that, despite substantial overlap between the ATS/JRS/ALAT guideline and the CHEST guideline, notable differences exist. The ATS/JRS/ALAT guideline prioritizes a probability-based diagnostic framework integrating exposure assessment, HRCT patterns, and BAL, whereas the CHEST guideline places greater emphasis on identifying a clear exposure and maintaining a higher threshold for diagnosis in its absence. Comparative analyses suggest that although this flexibility may improve diagnostic accuracy in real-world clinical settings, it may also increase the risk of confirmation bias, especially in cases without an identifiable antigen.^37^ Diagnostic uncertainty remains a major challenge in HP, particularly when no inciting antigen is identified. 

Differences between major guidelines also contribute to variability in clinical practice. These discrepancies may lead to divergent diagnostic classifications. In a prospective study, Buendia-Roldan et al.^38^ applied both diagnostic algorithms to 213 patients with confirmed HP to compare diagnostic confidence categorization. Using ATS/JRS/ALAT criteria, 18% of cases were classified as definite HP; 42% were classified as high confidence; 34% were classified as moderate confidence; and 4% were classified as low confidence. In contrast, the CHEST algorithm classified 65% as definite HP, 27% as high confidence, and 7% as low confidence. These findings highlight substantial variability in diagnostic likelihood assignment between the two guidelines, with CHEST criteria yielding a higher proportion of “definite” diagnoses.^38^

## TREATMENT

**Figure 4:**
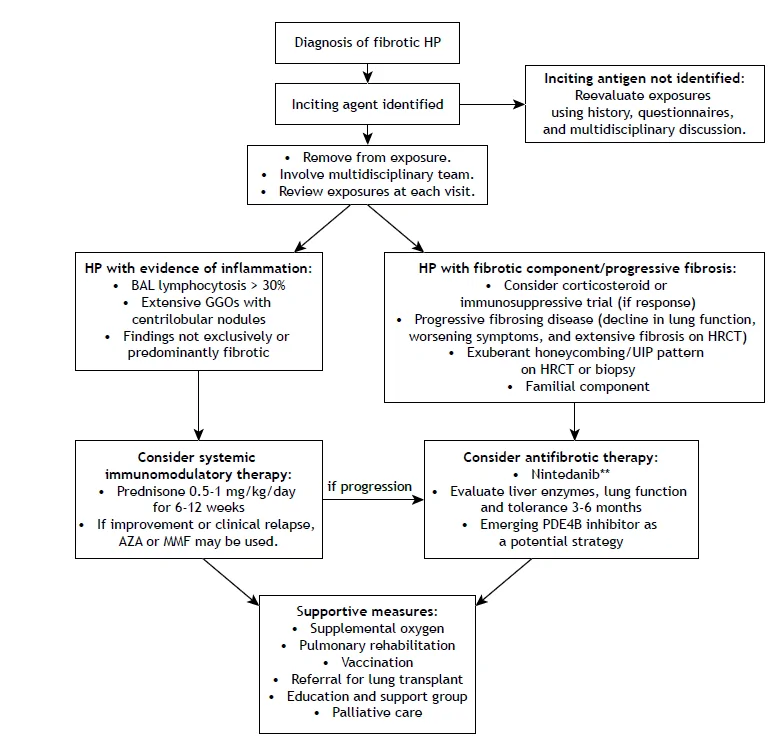
Flow chart for the management of hypersensitivity pneumonitis.* HP: hypersensitivity pneumonitis; GGOs: ground-glass opacities; UIP: usual interstitial pneumonia; AZA: azathioprine; MMF: mycophenolate mofetil; and PDE4B: phosphodiesterase 4B. *Adapted from Barnes & Johannson.^32^ **Although pirfenidone has shown potential efficacy in smaller studies and real-world cohorts of fibrotic HP, stronger evidence supports the use of nintedanib.

The management of HP encompasses a multidimensional approach that integrates antigen avoidance, pharmacological therapy, nonpharmacological interventions, and, in advanced cases, lung transplantation (Figure 4). In addition to elimination of the causative antigen, strategies such as corticosteroids and immunosuppressive agents may be considered in patients with active inflammation, and antifibrotic therapy has emerged as an option for those with progressive fibrotic disease. 

### Antigen avoidance

Causative antigens cannot be identified in 30-50% of patients with fibrotic HP, correct identification largely depending on the systematic use of environmental questionnaires, structured exposure assessments, in-home inspections, and antigen-specific testing. In many Latin American cohorts-particularly in Brazil-these standardized approaches are applied inconsistently, thus contributing to lower rates of confirmed exposure. Observational data consistently demonstrate that complete antigen avoidance is associated with better outcomes. In a cohort in the USA, complete avoidance eliminated recurrences and prevented progression to fibrosis, whereas incomplete avoidance was linked to relapse and subsequent fibrotic progression; in a multivariate analysis, ongoing exposure was also associated with a trend toward worse survival in patients with fibrotic disease.^26^ More recently, a large retrospective cohort study confirmed that antigen identification and strict avoidance determined survival benefits even in patients with fibrotic HP, with strict avoidance reducing the risk of death or lung transplantation by more than half. In contrast, patients with suspected but not avoided antigens and those with unidentified exposures had a more than twofold higher risk of adverse outcomes.^39^ Patients with fibrotic HP may be exposed to more than one antigen simultaneously or at different stages of the disease; however, the number and type of exposures do not appear to affect outcomes such as transplant-free survival.^40^

Recent reviews and guidance statements similarly emphasize the importance of implementing antigen avoidance wherever possible as the primary intervention, while acknowledging practical barriers (e.g., persistent avian antigen in homes despite bird removal) requiring aggressive remediation or relocation.^1^ ^,^ ^2^ ^,^ ^18^ ^,^ ^26^ ^,^ ^27^ ^,^ ^39^ Elimination of the antigen source is far more effective than reliance on personal protective equipment in all forms of HP. Examples include changing job roles or workplace settings in cases of occupational exposure and complete removal of birds, feathers, and droppings, along with deep cleaning of soft furnishings in cases of avian or mold-associated HP.^41^ The central challenge is that antigen avoidance depends not only on recognizing the exposure but also on the ability of patients to modify their environment or occupation. This creates disparities in treatment effectiveness, particularly in individuals unable to relocate, change jobs, or remediate their homes. 

The observation that strict avoidance improves outcomes even in patients with fibrotic HP reinforces the concept that antigen exposure continues to influence disease trajectory beyond the inflammatory and fibrotic phases. 

### Corticosteroids

Corticosteroids remain the most widely prescribed pharmacological therapy in patients with HP, particularly those with nonfibrotic forms, despite the fact that there is no robust study supporting its use (Chart 3). Prednisone is usually started at 0.5 mg/kg/day and tapered over weeks. The duration of treatment is variable. Several observational studies have shown that short-term corticosteroid therapy accelerates symptomatic relief and functional recovery, especially when combined with strict antigen avoidance. However, evidence for long-term benefit in preventing progression to fibrosis or improving survival is limited, and relapse is common if the inciting antigen is not eliminated.^41^ In a randomized trial of 36 patients with acute farmer’s lung, prednisolone improved DL_CO_ at 1 month but had no long-term effect on FVC or DL_CO_ at 5 years.^42^ In a retrospective cohort of fibrotic and nonfibrotic HP patients, corticosteroids and antigen avoidance improved lung function only in nonfibrotic patients, reversing monthly FVC decline from 0.35% to an increase of 0.84% (p < 0.001), with a nonsignificant DL_CO_ rise of 3.38%.^43^ The presence of BAL lymphocytosis (> 20%) has been associated with improvements in FVC with corticosteroid treatment.^43^

**Chart 3:** Pharmacological management of hypersensitivity pneumonitis.

Immunomodulatory therapy	
Prednisone	0.5-1 mg/kg/day for 6-12 weeks. More effective with BAL lymphocytosis > 30% or GGOs on HRCT scans.
Azathioprine	If improvement with corticosteroids or clinical relapse. Monitoring for hepatic and bone marrow toxicity.
Mycophenolate mofetil	Similar to azathioprine; potential improvement in DL_CO._
Antifibrotic therapy	
Nintedanib	Greater evidence supporting use in progressive disease. Limiting gastrointestinal adverse effects
Pirfenidone	Potential effect on disease progression. Evidence remains limited. Photosensitivity and gastrointestinal effects.
Nerandomilast	Anti-inflammatory and antifibrotic effects, with improved tolerability.

GGOs: ground-glass opacities.

In patients with fibrotic HP, the role of corticosteroids is more controversial. While corticosteroids may provide modest symptomatic relief or transient improvement in lung function, studies have reported limited efficacy in altering the progressive fibrotic course.^43^ Signs of active disease with ongoing inflammation may indicate a potential response to corticosteroid therapy, such as BAL lymphocytosis > 30%, extensive ground-glass opacities, nodularity, mosaic attenuation on HRCT scans, and findings that are not exclusively or predominantly fibrotic. The variable response to corticosteroids illustrates the importance of integrating radiological, physiological, and BAL data to determine whether a patient remains in an inflammatory phase amenable to treatment or has transitioned to an irreversible fibrotic stage. 

In practice, the decision to start or continue corticosteroids must therefore consider not only disease phenotype but also the risks of chronic therapy-particularly in older patients or those with telomere-related abnormalities-highlighting the need for early phenotypic classification and regular reassessment of treatment response. 

### Immunosuppression

Immunosuppressive agents may be used in patients with HP, particularly when there is response to corticosteroids and consideration of long-term use (Chart 3). Azathioprine and mycophenolate mofetil are the most frequently used, with observational studies suggesting potential benefits in improving or stabilizing lung function and reducing corticosteroid requirements. Retrospective series reported stabilization or modest improvements in FVC and DL_CO_ in patients receiving immunosuppressive therapy, especially those with nonfibrotic phenotypes.^44^ Immunosuppressants are associated with fewer metabolic and infectious complications than is prolonged corticosteroid exposure, supporting their role as maintenance or adjunctive therapy in selected patients.^45^

In patients with fibrotic HP, however, the efficacy of immunosuppressive therapy remains less certain. Although some cohort studies have suggested a slower decline in lung function with azathioprine or mycophenolate, others have found either no significant benefit or even potential harm when azathioprine or mycophenolate is used in combination with corticosteroids, as highlighted in studies investigating IPF.^46^ Other studies of fibrotic HP have shown disease progression despite the use of mycophenolate, azathioprine, or corticosteroids.^43^ ^,^ ^45^ Although cyclophosphamide and rituximab have been used in severe or rapidly progressive cases, evidence is limited to case series and small cohorts.^32^ ^,^ ^41^ ^,^ ^47^ Current guidelines emphasize that immunosuppressive therapy should be reserved for patients with evidence of ongoing inflammatory activity or frequent relapses, whereas antifibrotic agents may be preferable in those with predominantly fibrotic and progressive disease.^1^ ^,^ ^2^ Further analyses have shown that patients carrying genetic variants associated with shorter peripheral blood leukocyte telomere length have a higher risk of death, lung transplantation, functional decline, and hospitalization when treated with immunosuppressants such as mycophenolate in comparison with placebo-treated controls.^48^ ^,^ ^49^

### Antifibrotic therapy

The recognition that a substantial proportion of patients with fibrotic HP may follow a clinical course similar to that of those with PPF has led to increasing interest in antifibrotic therapy (Chart 3). Nintedanib and pirfenidone were initially developed for patients with IPF but have been evaluated in those with progressive fibrosing ILDs, including fibrotic HP.^50^ In a randomized, double-blind, placebo-controlled, parallel-group trial conducted at 153 sites in 15 countries, nintedanib significantly reduced the annual rate of FVC decline, with consistent benefits in the fibrotic HP subgroup.^3^

Pirfenidone has also shown potential efficacy in smaller studies and real-world cohorts of patients with fibrotic HP, with data suggesting attenuation of lung function decline and improvement in progression-free survival.^51^ However, the evidence remains less robust than that for nintedanib, and further randomized trials are warranted to clarify the role of pirfenidone in patients with fibrotic HP. Current guidelines recommend antifibrotic therapy as a treatment option for patients with progressive fibrotic HP, particularly when there is a predominant component of fibrosis and limited evidence of ongoing inflammation. The decision to initiate antifibrotic therapy should therefore be individualized, with disease behavior, radiological features, and prior response to immunomodulatory treatment being taken into consideration.^32^ ^,^ ^41^ ^,^ ^51^ Nerandomilast is a selective phosphodiesterase 4B inhibitor that has emerged as a potential therapeutic strategy in the management of HP patients presenting with a progressive fibrotic pattern. Recent studies have demonstrated benefits in subgroups of patients, including patients with fibrotic HP, with reductions in FVC decline over time and a tolerable adverse event profile.^52^ At the time of this publication, nerandomilast is still being evaluated in extension studies and has yet to be approved for use in Brazil by national regulatory agencies. The results of such extension studies are likely to be available soon. 

Antifibrotic therapy should not be interpreted as a replacement for antigen avoidance or immunomodulation but rather as a treatment strategy in patients whose disease progresses despite adequate exposure control and, when appropriate, as a trial of anti-inflammatory therapy. This integrated approach aligns with real-world clinical decision-making, where phenotype overlap and uncertainty are common. In practice, certain clinical features-such as persistent FVC decline, radiological progression characterized by traction bronchiectasis or reticulation, and limited response to corticosteroids-may help identify patients who derive the greatest benefit from antifibrotic therapy. Early recognition of this “fibrotic shift” is crucial because delays in initiating antifibrotic treatment may result in irreversible progression. 

### Other treatment modalities

Nonpharmacological interventions are essential in the management of HP. Preventive measures include vaccination against influenza, pneumococcus, respiratory syncytial virus, and SARS-CoV-2. Supplemental oxygen is recommended for patients with resting or exertional hypoxemia, with the goal of maintaining arterial oxygen saturation above 88-90%,^53^ and should be reassessed periodically. 

Pulmonary rehabilitation is indicated for patients with exercise limitation, persistent dyspnea, or impaired functional capacity, because it improves exercise tolerance, symptom burden, and health-related quality of life.^54^ In patients with advanced or progressive fibrotic HP, referral for lung transplantation should be considered in those with severe functional impairment (e.g., an FVC of < 50-60% of the predicted value and a DL_CO_ of < 40% of the predicted value), rapid decline in lung function, or long-term oxygen dependence. Early referral is recommended to optimize listing opportunities and improve post-transplant outcomes.^55^ Palliative care should also be considered in patients with advanced or progressive HP, focusing on symptom relief, quality of life, and multidisciplinary support, particularly when disease-modifying therapies are no longer effective or feasible.^56^

## PROGNOSIS AND FOLLOW-UP

In patients with HP, prognosis is largely determined by the presence of fibrosis and a PPF phenotype, which are linked to a greater and faster lung function decline and higher mortality than those observed in those with nonfibrotic disease. Factors associated with worse survival include an inability to remove the inciting antigen, older age, male sex, and a history of smoking.^26^ ^,^ ^57^ ^,^ ^58^ Lower BAL lymphocytosis is associated with reduced survival, likely because patients with higher BAL lymphocytosis tend to be those with a nonfibrotic pattern.^26^ ^,^ ^59^ The presence and extent of fibrosis, honeycombing, and traction bronchiectasis on HRCT scans are associated with a higher risk of mortality in patients with HP, whereas air trapping and mosaic attenuation are associated with greater survival.^28^ ^,^ ^29^ ^,^ ^33^ ^,^ ^58^ ^-^ ^60^ Specific histopathological features, such as an unusual interstitial pneumonia pattern, are also associated with a higher risk of mortality in patients with HP.^34^ Lower FVC or DL_CO_ or a decline in FVC reflect the progression of ILD and are associated with a greater risk of mortality, consistent with observations in other fibrosing ILDs.^26^ ^,^ ^34^ ^,^ ^57^ ^,^ ^58^

A variety of blood and BAL biomarkers have been investigated for their prognostic value in fibrotic HP, including serum Krebs von den Lungen-6 and surfactant protein D. However, none have shown consistent associations with the progressive phenotype.^61^ Further studies are needed to validate the role of these and other potential biomarkers, such as the eosinophil-to-lymphocyte ratio and peripheral blood monocyte count.^62^ Other conditions, such as the development of PH in ILD-including fibrotic HP-portend a markedly worse survival and greater morbidity, underscoring the need for early detection and, when indicated, targeted management.^33^ ^,^ ^45^ ^,^ ^58^

Patients with HP should be monitored for disease progression, including regular reviews of symptoms and pulmonary function tests every six months initially, and repeat HRCT scans annually as clinically indicated. 

## COMORBIDITIES

Comorbidities are common in patients with fibrotic ILD. The burden of comorbidities is also relevant and is linked to increased mortality in patients with HP.^64^ Group 3 PH stands out as particularly important because of its strong association with reduced exercise capacity, greater symptom burden, and poorer survival. The reported prevalence of PH in patients with HP ranges from 28.3% to 59%, with fibrotic patterns on HRCT scans and exertional hypoxemia identified as key predictors.^65^ ^-^ ^67^ In a recent study involving 90 patients with fibrotic and nonfibrotic HP, PH was present in 59% of cases, mostly exhibiting a mixed pre- and postcapillary component and showing that cardiovascular changes are also important factors in this population.^67^

The possibility of PH highlights the need for early screening, particularly in patients with unexplained dyspnea or disproportionate functional limitation, given that timely diagnosis allows targeted interventions and optimized management strategies.^58^ ^,^ ^64^ ^,^ ^68^ Moreover, patients with HP have a higher risk of other comorbidities, such as cardiovascular disease, hypertension, gastroesophageal reflux, diabetes, and coronary heart disease.^69^

In a prospective cohort of patients with fibrotic HP, patients presented with a high burden of comorbid conditions, the most common of which were cardiovascular disease, in 65%, hypertension, in 56%, and gastroesophageal reflux disease, in 24%.^64^ Although the overall number of comorbidities did not independently predict survival, specific conditions-particularly PH, diastolic cardiac dysfunction, and cerebrovascular disease-were significantly associated with increased mortality.^64^

Other comorbidities play an important role in HP and can impact quality of life and survival. Obstructive sleep apnea has a higher prevalence in patients with fibrotic ILD, potentially contributing to disease progression through repetitive microaspiration or intermittent hypoxemia. Osteoporosis related to long-term corticosteroid exposure and an increased risk of lung cancer in patients with fibrotic HP further complicate patient care.^57^ ^,^ ^58^ ^,^ ^64^

From a clinical perspective, the high burden of comorbidities in patients with HP reinforces the need for a comprehensive, multidimensional management strategy beyond ILD-directed therapy alone. Many of these comorbidities, particularly PH, cardiac dysfunction, gastroesophageal reflux disease, and sleep-disordered breathing, can amplify respiratory symptoms, mimic disease progression, or even accelerate functional decline. Distinguishing between HP progression and comorbidity-driven deterioration is therefore essential to guide appropriate treatment. 

## PERSPECTIVES

Future diagnostic strategies for HP are evolving toward earlier, more accurate, and biologically informed phenotyping. Beyond refined HRCT interpretation, machine learning-based image analysis is advancing, offering quantitative, reproducible patterns for fibrosis detection and progression monitoring.^70^ ^,^ ^71^ Concurrently, proteomic profiling is revealing novel plasma biomarkers linked to progressive fibrosing ILD, including HP, allowing derivation of a multiprotein signature with high sensitivity and negative predictive value for near-term progression.^63^ ^,^ ^71^ ^,^ ^72^ Integration of artificial intelligence-driven imaging analysis with multiomic biomarkers could transform multidisciplinary evaluation, enabling earlier and more precise diagnosis of HP subtypes and disease progression prediction. 

Antifibrotic agents such as nintedanib are now part of the HP therapeutic arsenal, and targeted agents such as the phosphodiesterase 4B inhibitor nerandomilast have shown promising results in slowing disease progression. In addition to these, proteomic insights may guide identification of novel molecular pathways amenable to therapy-such as integrin αvβ6, implicated in fibrogenesis and identified across progressive fibrosing subtypes.^52^ ^,^ ^72^ Management of HP should be increasingly guided by individualized approaches that consider patient-specific phenotypic characteristics and principles of personalized medicine. Distinct clinical, radiological, and immunological features can inform the choice and intensity of therapeutic interventions.^73^

Recent advances have also focused on the evaluation of PH-ILD, including HP. Randomized controlled trials testing inhaled treprostinil in patients with PH-ILD have shown improvement in the six-minute walk distance and delayed clinical worsening, leading to regulatory approval of this therapy.^74^ Recent data from an international registry including patients with fibrotic HP showed that, in those with severe hemodynamic impairment (pulmonary vascular resistance > 5 Wood units), treatment with phosphodiesterase 5 inhibitors was associated with improved outcomes.^75^

From a broader perspective, several gaps remain, particularly in Latin America. Epidemiological data on incidence, prevalence, and regional exposure profiles are limited, and research on environmental determinants, genetic susceptibility, and outcomes in diverse populations is still scarce. Addressing these unmet needs, as well as developing standardized approaches to exposure assessment and biomarker-driven diagnostics, should be priorities for future investigations. 

## REGIONAL CONTEXT AND CONSIDERATIONS IN LATIN AMERICA AND BRAZIL

In many Latin American countries, including Brazil, the path to diagnosing HP is hindered by structural and economic barriers. In a study comparing patient journeys in Brazil and Mexico, the authors found that patients are frequently referred to ILD centers with advanced disease stages because of limited access to HRCT, BAL, and lung biopsy techniques.^76^ In addition to diagnostics, the Brazilian and Latin American contexts impose challenges for treatment. 

Common environmental exposures in Brazil-such as mold and avian antigens, given the large number of captive birds in the country-likely contribute significantly to HP burden.^17^ However, access to antifibrotic therapy (e.g., nintedanib and pirfenidone) is limited in the public health care system. Although these medications are approved locally, their cost and distribution barriers prevent their widespread use, especially in publicly funded settings.^76^ As such, many Brazilian patients may not benefit from the antifibrotic and immunosuppressive therapies shown in trials, highlighting a critical gap between evidence and real-world care in the Latin American setting. 

## FINAL CONSIDERATIONS

In summary, HP remains a complex and heterogeneous disease that requires a multidisciplinary approach for accurate diagnosis and optimal management. Recent advances in imaging, histopathology, and molecular biomarkers-along with emerging tools such as artificial intelligence-are improving the recognition of fibrotic and nonfibrotic phenotypes and aiding in risk stratification. Early identification of progressive fibrotic forms and timely initiation of therapy are critical to slowing disease progression, whereas strict antigen avoidance and patient education remain fundamental across all presentations. Ongoing research into immune mechanisms, novel antifibrotic agents, and personalized treatment strategies holds promise for refining prognostic models and improving outcomes, highlighting the need for continued collaboration between clinicians, radiologists, and pathologists in clinical practice and future trials. 
